# A case report of eyelid Merkel cell carcinoma occurring under treatment with nivolumab for a lung adenocarcinoma

**DOI:** 10.1186/s12885-018-4919-z

**Published:** 2018-10-22

**Authors:** Daniele Lavacchi, Stefania Nobili, Marco Brugia, Agnese Paderi, Sara Fancelli, Enrico Caliman, Federica Vergoni, Enrico Mini

**Affiliations:** 1 School of Human Health Sciences, University of Florence, Largo Brambilla 3, 50134 Florence, Italy; 2 Department of Health Sciences, University of Florence, viale Pieraccini, 6, 50139 Florence, Italy; 3 Department of Experimental and Clinical Medicine, University of Florence, Largo Brambilla 3, 50134 Florence, Italy; 4 Pathological Anatomy Unit, Careggi University Hospital, Largo Brambilla 3, 50134 Florence, Italy

**Keywords:** Merkel cell carcinoma, NSCLC, Nivolumab, MCPyV, Eyelid MCC, Elderly

## Abstract

**Background:**

Merkel cell carcinoma (MCC) is a rare neuroendocrine malignancy of the skin characterized by high aggressiveness. Four main factors are implicated in its development: immunosuppression, ultraviolet radiation, age and the Merkel cell polyomavirus (MCPyV). In recent years, immune checkpoint inhibitors have shown clinical activity in MCC treatment.

**Case presentation:**

We report the case of an 82-year-old man with a lung adenocarcinoma diagnosis, who underwent immunotherapy with nivolumab as second-line treatment. Seven months after the diagnosis of lung cancer during the nivolumab treatment, the patient developed an eyelid MCC, initially misdiagnosed as a chalazion. A palliative radiotherapy was performed with clinical benefit. After a total of seven cycles of nivolumab, computed tomography showed a lung and cerebral disease progression. In addition, clinical conditions worsened leading to the patient’s death 13 months after the initial lung cancer diagnosis.

**Conclusions:**

Cases of co-occurrence of MCC and non-small cell lung cancer (NSCLC) have rarely been reported. Interestingly, common risk factors may be postulated for both cancers. Considering the rarity of this adverse event, its short-term temporal relation with the administration of the drug, which makes a relation improbable, and the coexistence of other risk factors, which may provide plausible explanations, it is possible to conclude according to the WHO Adverse Reaction Terminology that a causal relation between the occurrence of this serious adverse event and the exposure to the drug is unlikely. However, the case deserves to be reported in the literature.

## Background

Merkel cell carcinoma (MCC) is a rare neuroendocrine malignancy of the skin characterized by a high aggressiveness with an overall survival of 10 months in the metastatic setting [1, 2]. It is an age-related cancer with a higher incidence in elderly patients. Historically, the two main factors implicated in the onset of MCC were exposure to ultraviolet rays and immunosuppression [3]. In 2008 Feng et al. revealed a new polyomavirus, until then unknown, in MCC tissue samples - the Merkel cell polyomavirus (MCPyV) [4]. Several studies have shown that MCPyV-DNA is integrated into tumor cells in about 80% of MCC cases, inferring that infection plays an important role in the pathogenesis of MCC [4, 5].

About a half of all MCCs originate from the head and neck (H&N) area. 5–20% of the H&N MCCs originate in the eyelids [6]. In most cases, the lesion is rapidly evolving and the diagnosis is not always readily identified. In fact, the lesion is often misdiagnosed as a chalazion or a stye [6, 7].

MCC shows very low response rates to cytotoxic chemotherapy [8–10]. In recent years, immune checkpoint inhibitors such as avelumab, an anti-programmed death ligand 1 (PD-L1) monoclonal antibody (MoAb), pembrolizumab and nivolumab, anti-programmed death 1 (PD-1) MoAbs, have shown clinical activity in the treatment of MCC. [11–16]. On March 23, 2017, the U.S. Food and Drug Administration granted accelerated approval to avelumab for the treatment of patients with metastatic MCC [11].

## Case presentation

We report the case of an 82-year-old man, who underwent a total body computed tomography (CT) on February 2017, due to the occurrence of cough. CT showed an extensive mass in the left upper lobe of the lung. Thus, a bronchoscopy with transbronchial needle aspiration (TBNA) was performed. The cytological examination was compatible with lung adenocarcinoma. Epidermal growth factor receptor (EGFR) mutations and anaplastic lymphoma kinase (ALK) translocation were tested to determine the most appropriate treatment but no mutation was detected. It was not possible to test PD-L1 expression because only cytological samples were available.

To complete the staging of the disease, the patient underwent a positron emission tomography (PET) examination. PET showed a massive tracer uptake at the pulmonary mass and showed an extensive involvement of the hilar and mediastinal lymph nodes. Before starting the treatment, a further TC scan was performed in May. TC showed an increased pulmonary mass involving approximately the entire left lung.

The patient referred a smoking history and as comorbidities: arterial hypertension, osteoporotic and traumatic vertebral fractures, iatrogenic bone marrow lesion resulting from surgery for discopathy, benign prostatic hypertrophy treated with transurethral resection, pulmonary emphysema, carotid vasculopathy and abdominal aneurysm. He had an ECOG performance status of 2.

In relation to clinical conditions, age and comorbidities, the patient underwent two chemotherapy cycles with oral vinorelbine (day 1,8 every 21), the latter of which was administered in July. During the treatment, the patient experienced fatigue G1, diarrhea G1, constipation G1, anorexia G1 and hyperkinetic supraventricular arrhythmia treated with amiodarone.

The restaging CT was performed in July and showed lung disease progression. Thus, from July to December, the patient received 3 mg/kg nivolumab (day 1 every 14) as second-line treatment for a total of seven cycles. Based on body weight, nivolumab was administered at a dose of 195 mg for the first two cycles and 205 mg for the subsequent five cycles. After the first two doses, the patient was hospitalized at the emergency department with a diagnosis of pneumonia. Therefore, antibiotics and corticosteroids were administered and a clinical improvement was obtained. In September, the patient recovered a good respiratory performance, so he was able to restart nivolumab. The subsequent five cycles were well tolerated except for grade 1 hypothyroidism and grade 2 fatigue.

**Fig. 1:**
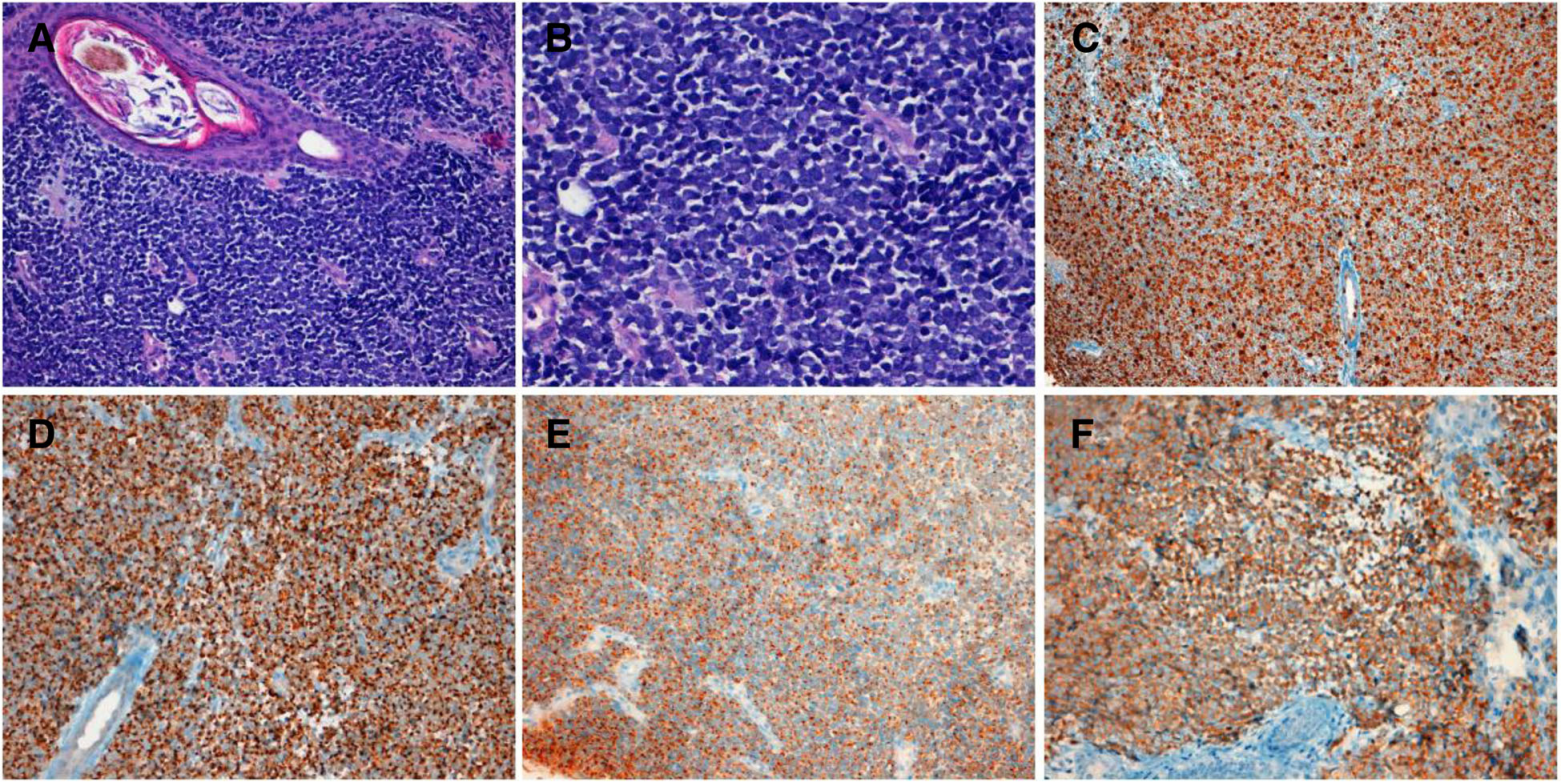
Histological examination. **a** Hematoxylin-eosin (20×); **b** Hematoxylin-eosin (40×); **c** Ki67 (10×); **d** Cytokeratin 20 (20×); **e** Chromogranin (20×); **f** Synaptophysin (20×)

Collaterally, in September, the patient showed a small nodular dome shaped lesion on the upper eyelid. He consulted a private ophthalmologist who initially suspected a diagnosis of chalazion. However, the lesion increased until about 3 cm within a few weeks. Thus, the patient came back to the Ophthalmology Department of Azienda Ospedaliera-Universitaria Careggi of Florence where a biopsy was performed, with the suspect of a malignant lesion. The histological examination indicated an MCC (immunohistochemical study: cytokeratin 20+, synaptophysin+, chromogranin+, CD20-, CD3-, Ki67 60–70%) (Fig. 1).

A nuclear magnetic resonance imaging of the orbit and neck did not show lymph node involvement.

The case was collegially discussed. Since the patient was at high risk for anesthesia due to his clinical conditions and given the prognosis of lung cancer, surgery was contraindicated. Thus, a palliative radiotherapy was proposed and was started in December when the lesion had expanded to about 5 cm in the anteroposterior diameter. Following radiotherapy, the size of the eyelid lesion was reduced to a few millimeters.

In December 2017, after a total of seven cycles of nivolumab, a CT scan was also performed. It showed a lung and cerebral disease progression. In addition, there was a worsening of clinical conditions with increased cough and fatigue. Therefore, the best supportive care was provided until the patient died in March 2018.

## Discussion and conclusions

Our patient developed an eyelid MCC 7 months after the diagnosis of lung cancer. Available data in literature shows that, overall, there is no statistically significant risk to develop an MCC following a diagnosis of primary lung cancer (i.e. standardized significant rate (SIR) 0.88; 95% CI 0.32–1.92), as well as to develop an MCC within 1 year from the diagnosis of lung cancer ((SIR) 0.63; 95% CI 0.13–1.85) [17].

The development of MCC occurred during a treatment with nivolumab for a lung adenocarcinoma. Nivolumab is a fully human IgG4 immune checkpoint inhibitor antibody targeting PD-1 that has been approved for the treatment of advanced or metastatic non-squamous non-small-cell lung cancer (NSCLC) according to positive results of the phase III, open label study CheckMate 057 [18].

An analysis of the literature was performed to know whether any MCC occurrences in the course of a nivolumab treatment had been reported. The research did not identify any publications related to the occurrence of MMC during nivolumab treatments. Based on post-marketing reports of the nivolumab producer, one case of neuroendocrine carcinoma during the treatment with this drug for lung adenocarcinoma has been described. However, since this type of event is reported voluntarily from a population of uncertain size, it is generally not possible to reliably estimate the frequency or to establish a causal relationship to the drug exposure.

MCC represents a checkpoint inhibitor responsive cancer. Factors that have been suggested to contribute to an immunotherapy response are represented by a high tumor mutational burden due to the ultraviolet rays exposure [19] and the integration of MCPyV DNA sequence. In fact, high expression levels of inhibitory receptors such as PD-1 and Tim-3 have been found on MCC infiltrating lymphocytes and T CD8-specific MCPyV cells [5]. These factors offer a rationale for treating patients with anti PD-1/anti PD-L1 monoclonal antibodies.

Avelumab is indicated in the treatment of MCC based on an open-label, single-arm, multi-center clinical trial (JAVELIN Merkel 200 trial). It induced an objective response (OR) in 33% of treated patients [11]. Responses to avelumab were observed irrespective of PD-L1 expression on tumor cells or Merkel cell polyomavirus status [12].

In a multicentre phase 2 noncontrolled trial including 26 patients with an advanced MCC pembrolizumab induced an OR in 56% of patients. PD-L1 expression was more frequent in MCPyV-positive cancers than in MCPyV-negative cancers (71% vs 25%). Responses were observed in both virus-positive and virus-negative. No significant correlation of PD-L1 expression on tumor cells or infiltrating immune cells with drug response was observed [13].

A significant and durable response in a patient with metastatic MCC following nivolumab therapy was reported [14]. Preliminary results of an ongoing non-comparative, multiple cohort, open-label, phase 1/2 study (CheckMate 358) of nivolumab in patients (*n* = 50 MCC) with virus-positive and virus-negative solid tumors show an OR in 64% of advanced MCC cases and pathologic tumor regressions in 65% of resectable MCC cases [15, 16]. Moreover, a phase 2 clinical trial evaluating nivolumab + ipilimumab +/− stereotactic body radiation therapy (SBRT) for metastatic MCC is ongoing (NCT03071406).

Four main factors are implicated in the development of MCC: immunosuppression [20–25], ultraviolet radiation [3], age [26], and MCPyV [4, 5]. Overall, our patient presented three out of four MCC risk factors, whereas the MCPyV positivity status was unknown.

As far as immunosuppression is concerned, it is known that immunosuppressed patients have a poor prognosis with a reduced MCC-specific survival [20]. Immunosuppressive conditions determining an increased risk of developing an MCC are: patients with haematological malignancies, especially chronic lymphocytic leukaemia [21, 22], acquired immunodeficiency syndrome (AIDS) [23], patients undergoing organ transplantation [24] or patients in chronic treatment for autoimmune diseases [25]. Our patient did not have an immunosuppressive state such as those described above, but he had a lung cancer and he previously had received chemotherapy with vinorelbine.

It is also well known that there is a strong association between MCC and a history of sun exposure [3]. Ultraviolet-associated mutations in RB1, TP53, NOTCH1 and FAT1 genes have been reported in MCPyV-negative cancers [19]. In our patient, the MCC developed in one of the most sun-exposed anatomical sites, so prolonged exposure to ultraviolet light may have been a relevant causal factor of the MCC. On the basis of the information gained by the anamnestic collection, a history of high sun exposure both before and during the treatments was highlighted.

Regarding age, it has been shown that H&N MCCs have a higher incidence in elderly patients. It is known that MCPyV seroprevalence increases according to age achieving 73% in subjects older than 70 years [26].

Our patient had two cancers in which MCPyV has been proposed to be related with the pathogenesis. In fact, several studies have reported the presence of MCPyV-DNA integrated into tumor cells in about 80% of MCC cases [4, 5] and in a range from 9 to 18% in NSCLCs [27–29]. Thus, although the underlying tumorigenic mechanisms are unknown, in a subset of patients MCPyV could play an important role also in the pathogenesis of NSCLC, through the expression of two putagenic oncoproteins (i.e. large T and small T antigens) [29, 30]. Unfortunately, we were not able to determine the presence of MCPyV DNA due to the unavailability of biological material. However, it must be noted that although MCC is a rare cancer, MCPyV is ubiquitous in human populations (i.e. from 60 to 80% of the general population is infected with MCPyV) [30].

In conclusion, we described a case of eyelid MCC occurring in a patient with recent diagnosis of NSCLC during second-line treatment with nivolumab. Cases of co-occurrence of MCC and NSCLC have rarely been reported. Interestingly, common risk factors may be postulated for both cancers. Considering the rarity of this adverse event, its short-term temporal relation with the administration of the drug, which makes a relation improbable, and the coexistence of other risk factors, which may provide plausible explanations, it is possible to conclude according to the WHO Adverse Reaction Terminology [31] that a causal relation between the occurrence of this serious adverse event and the exposure to the drug is unlikely. However, the case deserves to be reported in the literature.

## Abbreviations

AIDS: Acquired immunodeficiency syndrome
ALK: Anaplastic lymphoma kinase
CT: Computed tomography
EGFR: Epidermal growth factor receptor
H&N: Head and neck
MCC: Merkel cell carcinoma
MCPyV: Merkel cell polyomavirus
MoAb: Monoclonal antibody
NSCLC: Non-small-cell lung cancer
OR: Objective response
PD-1: programmed death 1
PD-L1: Programmed death ligand 1
PET: Positron emission tomography
SBRT: Stereotactic body radiation therapy
SIR: Standardized significant rate
TBNA: transbronchial needle aspiration
WHO: World health organization
